# Editorial: Aquatic nutrition and intestine immunity

**DOI:** 10.3389/fimmu.2023.1274213

**Published:** 2023-09-12

**Authors:** Qiyou Xu, Mingchun Ren, Liansheng Wang, Fernando Y. Yamamoto, Changle Qi

**Affiliations:** ^1^ College of Life Science, Huzhou University, Huzhou, China; ^2^ Freshwater Fisheries Research Center, Chinese Academy of Fishery Sciences, Jiangsu, China; ^3^ Heilongjiang River Fisheries Research Institute, Chinese Academy of Fishery Sciences, Harbin, Heilongjiang, China; ^4^ Thad Cochran National Warmwater Aquaculture Center, Mississippi Agriculture and Forestry Experiment Station, Mississippi State University, Stoneville, MS, United States; ^5^ Department of Wildlife, Fisheries and Aquaculture, Mississippi State University, Starkville, MS, United States

**Keywords:** nutrition, feed additive, intestine, immunity, aquatic animal

Aquaculture supplies large quantities of high-value proteins for human consumption (1). Due to the limited supply of fishmeal and the increasing demand of aquaculture worldwide, novel protein ingredients are being widely adopted for their lower cost and sustainability as a fish meal replacement in the feed industry (2). Previous studies have shown that these alternative proteins can partially replace fishmeal in the diets of fish. However, excessive inclusion levels of these novel proteins may result in impair growth performance, induce liver inflammation, and compromise the intestinal structure of farmed fish (3). It is of great importance to explore functional nutrients to improve immunity and maintain health of different aquatic animals. The intestinal tract is the main site for digestion and absorption of nutrients, and it is crucial for fish growth and physiological functions. Therefore, it is of utmost importance to investigate the fish intestinal health when fed low fish meal diets and develop protocols to mitigate potential side effects caused by the inclusion of alternative protein ingredients.

The goal of this Research Topic is to provide literature on advanced research and improving our understanding of the nutritional immunity regulation in multiple aspects of aquatic animals on intestine. Areas covered include: 1) Effects of replacing fishmeal with novel proteins on growth performance and intestinal health; 2) Effects of supplements on growth, intestine immunity of aquatic animal fed with a low-fishmeal diet; 3) Dietary supplementation of additives to improve intestinal health.

The global fishmeal production has been stagnated for decades and cannot keep up with the increased demand by aquaculture; Thus, fishmeal price has significantly increased over the years (4). It is important to search for alternative protein sources to reduce fishmeal levels in the diet. Soybean meal is the most common fishmeal substitute in aquafeed due to its steady supply and relatively balanced amino acid profile (5). But long-term feeding of soybean meal may result in structural and functional changes in the distal intestine (6). The negative influences may be correlated with antinutritional factors in soybean meal (7). Wang et al. reported that diet supplementation with 2.46% soybean saponin not only hindered the growth performance by negatively affecting the macronutrients absorption in the intestine, but also induced an inflammatory response in the large intestine possibly by damaging the intestinal morphology, disrupting the intestinal microbiota and decreasing intestinal epithelial cell membrane permeability. Luo et al. reported that dietary soybean meal compromised the intestinal health, and the adverse effects were related to the presence of β-conglycinin and glycinin, especially glycinin. Cottonseed protein concentrate (CPC) is a novel non-food protein derived from cottonseed. Wang et al. replaced fishmeal with 15% CPC and largemouth bass even exhibited better growth potential during the last three weeks of whole feeding trial, which was accompanied with higher phosphorylation level of TOR signaling and higher mRNA expression level of myogenin (myog). However, largemouth bass fed with CPC presented higher inflammation in both liver and gills during *N. seriolae* infection challenge. Liu et al. showed no adverse effects on growth performance of juvenile rainbow trout (*Oncorhynchus mykiss*) when 75% dietary fishmeal was replaced by CPC. Nevertheless, the CPC-based diet resulted in reduced the activity of intestinal trypsin, decreased villus height and width in the distal intestine, upregulated mRNA expression levels of inflammatory cytokines in the intestine, and impaired gut microbiota with reduced bacterial diversity and decreased the relative abundance of *Bacillaceae*. *Clostridium autoethanogenum* protein (CAP) is a byproduct of *Clostridium autoethanogenum* fermentation to produce ethanol. Chen et al. reported that dietary CAP could improve the growth, disease resistance, digestive capacity, and modulate the intestinal microbiota of *L. vannamei* with a higher immune response and enhanced the ability of shrimp to cope with salinity stress.

Previous studies have shown that appropriate inclusion of functional supplements in aquatic diets can effectively mitigate the adverse effects of low-fishmeal diets on farmed fish (8). Zheng et al. reported that CPC substitution induced a significant decrease in digestive enzyme activities and gut barrier protein PT-1 expression and a significant increase in g-GT enzyme activity and inflammatory related factors (Relish and Toll) expression of *Macrobrachium rosenbergii*, and B. coagulans could mediate specific gut microbes and the combined action of multiple functional secondary metabolites to affect intestinal barrier function, digestion, and inflammation. Liu et al. reported that 1.2% taurine supplementation in diets greatly enhanced the weight gain of juvenile golden pompano fed a low fishmeal diet. Moderate exogenous taurine increased the muscular thickness and villus length within the intestine, maintained intestinal physical barrier stability, activated the Nrf2/Keap-1/HO-1 signaling pathway, suppressed NF-kB signaling and intestinal pro-inflammatory cytokine gene expression, and increased anti-inflammatory cytokine gene expression. Liu et al. also reported that dietary cysteine (the precursor of taurine) greatly increased the SGR of golden pompano, upregulated the Nrf2/Keap1/HO-1 signaling pathway, improved muscle thickness and villi length, increased the diversity and relative abundance of the intestinal flora of golden pompano. Supplementation of cysteine also suppressed intestinal NF-kB/IKK/IkB signaling and pro-inflammatory cytokine mRNA levels. Conversely, intestinal anti-inflammatory cytokine gene expression and serum immune parameters were upregulated. Zhao et al. reported 1%-2% alanyl-glutamine and 0.1%-0.2% tributyrin can alleviate enteritis caused by high inclusion levels of soybean meal. The activities of intestinal trypsin, lipase and amylase in tributyrin and alanyl-glutamine groups increased significantly, and the gene expression levels of acetyl-CoA carboxylase (ACC), caspase-3, caspase-8, caspase-9, tumor necrosis factor alpha (TNF-α), and interleukin-1 beta (IL-1b) were down-regulated, while the gene expression level of target of rapamycin (TOR) and eIF4E-binding protein (4E-BP) were up-regulated.

Another aspect of interest, is the functional feed additives as an alternative prophylactic approach to improve animal health and performance (Rawling et al.). Porter et al. established rainbow trout cell lines as potential alternative method to test functional feed ingredients, GALT leucocytes were deemed most effective to act as a health screen over the 4 hr time point demonstrating responses to Poly I:C, PHA, and rIL-1b. RTS11 and RTgutGC also responded to the stimulants but did not give a strong T-cell response, most likely reflecting the nature of the cell type as opposed to the mixed cell populations from the primary GALT cell cultures. When stimulated with both forms of β-glucan, GALT leucocytes of rainbow trout demonstrated a strong proinflammatory and T-cell response. Zhang et al. reported that fish fed the diet with 300 mg kg^-1^β-glucan significantly increased the activity of lysozyme. Transcriptome analysis showed that 109 immune-related genes were differentially expressed. 300 mg kg^-1^ β-glucan significantly increased the relative abundance of *Mycoplasma* and decreased *Proteobacteria* (mainly *Escherichia-Shigella* and *Escherichia coli*) and *Bacillus anthracis* in largemouth bass intestinal microflora. Shen et al. suggested that β-1,3-glucan supplementation improved the intestinal health of white shrimp (*Litopenaeus vannamei*) through the modulation of intestinal microbiota, the suppression of intestinal inflammatory responses, the elevation of immune and antioxidant capacity, and promoted growth performance of white shrimp. The yeast cell wall (YCW) is an established prebiotic. Rawling et al. identified α-mannan content as a potent driver of GCD and IEL hyperplasia, suggestimg the fortification of intestinal barrier integrity and immune competence. Further the structural molecular differences of the yeast cell wall polysaccharides, in terms of and β-1,3-glucans, were shown to modify the expression pattern of PRR responses. Zhou et al. reported that oral administration of recombinant hepcidin improved the growth performance and regulated the iron metabolism. The immunity and survival of grass carp were improved, and hepcidin in combination with chitosan was better than that of hepcidin alone. Yang et al. suggested that tea polysaccharides promoted immunity, antioxidant capacity and intestinal barrier function and reduced lipogenesis and glucose transporter of common carp. Lu et al. reported mannose oligosaccharide (MOS) enhances immunity partly related to increasing antibacterial substances content and antimicrobial peptides expression. MOS attenuates inflammatory response partly related to regulating the dynamic balance of intestinal inflammatory cytokines. MOS regulates immune barrier function may partly be related to modulating TLRs/MyD88/NFkB and TOR/S6K1/4EBP signaling pathways. Luo et al. reported a-ketoglutarate may regulate intestinal energy via tricarboxylic acid cycle, thereby alleviating the damage intestinal morphology induced by the dietary soybean antigen proteins.

The underlying regulatory mechanism in aquatic animal nutrition and intestine immunity remains largely unresolved. This Research Topic showcases a collection of original research that highlights the latest discoveries and advances in the field of aquatic animal nutrition and intestinal immunity. By improving our understanding of the nutritional immunity regulation in multiple aspects of aquatic animals, there is potential to develop functional feeds and assist to the infinite growth potential of the aquaculture industry.

## Author contributions

QX: Writing – original draft, Writing – review & editing. MR: Conceptualization, Writing – review & editing. LW: Conceptualization, Writing – review & editing. FY: Writing – review & editing. CQ: Writing – review & editing.
